# Comparing Transactional eHealth Literacy of Individuals With Cancer and Surrogate Information Seekers: Mixed Methods Study

**DOI:** 10.2196/36714

**Published:** 2022-09-28

**Authors:** Taylor S Vasquez, Carma L Bylund, Jordan Alpert, Julia Close, Tien Le, Merry Jennifer Markham, Greenberry B Taylor, Samantha R Paige

**Affiliations:** 1 College of Journalism and Communications University of Florida Gainesville, FL United States; 2 College of Medicine University of Florida Gainesville, FL United States; 3 Division of Hematology & Oncology College of Medicine University of Florida Gainesville, FL United States; 4 Department of Communication Flagler College St. Augustine, FL United States; 5 Johnson & Johnson, Inc, Health & Wellness Solutions New Brunswick, NJ United States

**Keywords:** eHealth literacy, cancer communication, individuals with cancer, surrogate seekers, web-based information credibility appraisal

## Abstract

**Background:**

The number of adults entering higher-risk age groups for receiving a cancer diagnosis is rising, with predicted numbers of cancer cases expected to increase by nearly 50% by 2050. Living with cancer puts exceptional burdens on individuals and families during treatment and survivorship, including how they navigate their relationships with one another. One role that a member of a support network may enact is that of a surrogate seeker, who seeks information in an informal capacity on behalf of others. Individuals with cancer and surrogate seekers often use the internet to learn about cancer, but differences in their skills and strategies have received little empirical attention.

**Objective:**

This study aimed to examine the eHealth literacy of individuals with cancer and surrogate information seekers, including an investigation of how each group evaluates the credibility of web-based cancer information. As a secondary aim, we sought to explore the differences that exist between individuals with cancer and surrogate seekers pertaining to eHealth literacies and sociodemographic contexts.

**Methods:**

Between October 2019 and January 2020, we conducted a web-based survey of 282 individuals with cancer (n=185) and surrogate seekers (n=97). We used hierarchical linear regression analyses to explore differences in functional, communicative, critical, and translational eHealth literacy between individuals with cancer and surrogate seekers using the Transactional eHealth Literacy Instrument. Using a convergent, parallel mixed methods design, we also conducted a thematic content analysis of an open-ended survey response to qualitatively examine how each group evaluates web-based cancer information.

**Results:**

eHealth literacy scores did not differ between individuals with cancer and surrogate seekers, even after adjusting for sociodemographic variables. Individuals with cancer and surrogate seekers consider the credibility of web-based cancer information based on its channel (eg, National Institutes of Health). However, in evaluating web-based information, surrogate seekers were more likely than individuals with cancer to consider the presence and quality of scientific references supporting the information. Individuals with cancer were more likely than surrogate seekers to cross-reference other websites and web-based sources to establish consensus.

**Conclusions:**

Web-based cancer information accessibility and evaluation procedures differ among individuals with cancer and surrogate seekers and should be considered in future efforts to design web-based cancer education interventions. Future studies may also benefit from more stratified recruitment approaches and account for additional contextual factors to better understand the unique circumstances experienced within this population.

## Introduction

### Overview

The number of adults entering higher-risk age groups for receiving a cancer diagnosis is rising, with predicted numbers of cancer cases expected to increase by nearly 50% by 2050 [1]. Living with cancer puts exceptional burden on individuals and families during treatment and survivorship, including how they navigate their relationships with one another [2]. To best support individuals with cancer and members of their support networks with evidence-based programs, a better understanding of the unique needs and roles of each group is warranted [3].

Active participation in health care decision-making leads to better outcomes and increased quality of life and helps patients receive appropriate and cost-effective treatments [4]. Unfortunately, support networks tend to underassess their quality of life, bringing into question the understanding this population has of the perspective of an individual with cancer [5]. Informed support networks are better able to provide productive support, assist in treatment compliance, and help ensure the continuity of care for the patient [6]. Similarly, well-informed individuals with cancer who participate in shared decision-making with their clinicians have more positive cognitive outcomes related to their care [7,8].

One way in which individuals with cancer and support networks serve active roles in health care experiences is web-based health information seeking [9]. Blogs and social media have evolved past 1-way information websites into expansive, 2-way communicative resources that help individuals with cancer and caregivers connect and learn from other people’s experiences and expertise [10]. The availability of health and medical information through web-based forums and sources has increased dramatically over the last several decades and has created an abundance of opportunities for patients and support networks to engage in seeking health information [11,12]. Members of a support network who search for information pertaining to their family member or friend’s health and diagnosis are called *surrogate seekers* [11]. The act of surrogate seeking is defined as seeking “information in a nonprofessional or informal capacity on behalf (or because) of others without necessarily being asked to do so” [13]. Although these groups frequently use the internet to search for health information, the general population unfortunately has a challenging time evaluating the quality and veracity of web-based health information [14], and research has demonstrated that this challenge is evident among patients living with chronic ailments such as cancer [10]. Web-based cancer misinformation is prevalent and has the potential to cause harm to its consumers [15]. Investigating how individuals with cancer and surrogate seekers navigate web-based cancer information is imperative to begin developing interventions that support each group in health decision-making.

### Transactional eHealth Literacy

eHealth literacy is a dynamic, intrapersonal skill set that is shaped by the experiences, technologies, and opportunities available to an individual at a given time [16]. Paige et al [17] defined transactional eHealth literacy as, “the ability to locate, understand, exchange, and evaluate health information from the internet in the presence of dynamic contextual factors, and to apply the knowledge gained for the purposes of maintaining or improving health.” eHealth literacy is an expanding field of research, as a large portion of health-related messages and information is circulated and accessed through web-based, media, and digital sources [18] eHealth literacy requires combining both knowledge and skills from a diverse set of domains and is inherently and increasingly relevant to scholars, patients, and other individuals [18]. The Transactional Model of eHealth Literacy (TMeHL) centralizes the concept of communication within the context of how web-based health information is accessed, evaluated, and applied to inform health decisions. The TMeHL functions under a broad assumption that the ability of an individual to counteract challenges during the web-based experience is a continuous process, and the process is constantly modified according to diverse eHealth contextual factors and prior eHealth experiences. Transactional eHealth literacy includes the following four competencies: (1) functional (ie, the ability to locate and understand web-based information); (2) communicative (ie, the ability to exchange information among individuals within web-based contexts); (3) critical (ie, the ability to appraise and evaluate the source and content of information found on the web); and (4) translational (ie, the ability to use information learned from the internet to inform health care decisions) skills [19]. eHealth literacy is associated with sociodemographic and psychosocial variables. For example, people who are younger, report higher education, and use electronic devices more frequently have higher eHealth literacy than their counterparts [17,20]. A systematic review found that caregivers generally have higher health literacy levels than patients [21], but the eHealth literacy skills of patients with cancer and caregivers have received less attention. Caregivers are more likely to be female and younger, so differences are assumed [22], but confirmatory research is needed.

Sillence et al [23] reported that patients and caregivers have different web-based information needs and uniquely engage with website content to determine its quality and relevance. A report by the Pew Research Center [24] found that caregivers are more likely to consult web-based rankings of clinicians and medical facilities and use web-based reviews of drugs and medical treatments, whereas patients seek information about their diagnosis, the causes and spread of cancer, treatment options, and the side effects of treatment. There remains a lack of evidence regarding the specific cues and processes that patients and surrogate seekers use to evaluate the credibility, or the trust and quality, of web-based health information.

### Critical eHealth Literacy

Critical eHealth literacy is defined as the knowledge and ability of a person to evaluate the credibility, relevance, and risk of exchanging web-based health information [17]. The perceived credibility of health information is associated with the recipient’s satisfaction with the information found and is ultimately linked to proactive behavior change [23,25]. There is much debate about how to conceptualize credibility [26], but it is generally dependent on whether a consumer considers a piece of information to be believable or guided by trustworthiness and expertise [27]. When evaluating web-based health information, the public is encouraged to consider the source of the information and rely on educational or government agencies to cross-reference the content with other reliable sources and to consider the date on which the content was published, among other indicators [28]. Examining how patients and surrogate seekers assess the credibility of web-based cancer information could provide valuable insights into how lay audiences operationalize the concept of credibility. Such evidence will be valuable for the design and consistent evaluation of patient education resources that are developed to be perceived as credible by their recipients. Furthermore, this evidence will inform how to best deliver education from diverse sources within web-based contexts and offered by offline contacts (eg, clinicians and support networks).

### Purpose

This study aimed to evaluate the eHealth literacy of individuals with cancer and surrogate seekers. Given that perceived skills to evaluate web-based health information do not always translate into proficient performance behaviors [29], we also asked individuals with cancer and surrogate seekers to reflect on the processes they use to evaluate web-based cancer information.

- Hypothesis 1: compared with individuals with cancer, surrogate seekers will have a higher self-reported ability related to functional, communicative, critical, and translational eHealth literacy.
- Research question 1: What differences exist between individuals with cancer and surrogate seekers pertaining to eHealth literacy and sociodemographic contexts?
- Research question 2: What processes do individuals with cancer and surrogate seekers use to evaluate the credibility of web-based cancer information?

## Methods

### Recruitment

Between October 2019 and January 2020, we conducted a 20-minute web-based survey with adults registered with the broad consent research registry of a large southeastern medical university. The broad consent research registry is a database of individuals with cancer who volunteered to be contacted about research opportunities. We were provided with contact information for individuals with cancer who had consented to be contacted for research and who also had an International Classification of Diseases-10 code identifying cancer. Identified individuals with cancer (N=6847) were sent an email invitation, receiving up to 1 reminder. All identified individuals with cancer received a follow-up email because of our not distributing individualized survey links. This allowed potential participants to forward the email to other eligible individuals. The eligibility criteria included being an English-speaking adult (aged ≥18 years) and having used the internet to look for advice or information about (1) their own cancer or (2) a family member or friend’s cancer in the past 6 months. Surrogate seekers were identified through a snowball sampling technique. We sent an email invitation to the identified individuals with cancer:

If you have not searched for cancer information in the past six months but have a family member or friend that searches online cancer information for you, we would like to hear from them. Please consider forwarding them this email.

We did not identify dyads of patients and their support networks to examine similarities, differences, or trends. This study recruited surrogate seeker participants through referrals from individuals with cancer who completed the survey, but we did not establish any dyadic connections between patients and the surrogate seekers they recommended to complete the survey.

The participants who completed the survey were remunerated with a US $25 e–gift card for their time. The study data were collected and managed using REDCap (Research Electronic Data Capture; Vanderbilt University) tools hosted at the University of Florida [30]. REDCap is a secure, web-based application designed to support data capture for research studies. This manuscript reports a secondary analysis of items from the larger survey.

### Measures

The sociodemographic characteristics for this study, including age, sex, race, and socioeconomic status, were measured using items adopted from the Health Information National Trends Survey and the US Census Bureau (Multimedia Appendix 1). Participants indicated whether they were a person living with their own cancer diagnosis (ie, a patient). If not affirmed, participants were asked if they had used the internet to look for advice or information about someone else’s cancer. If they said yes, they were included in the study as a surrogate information seeker.

We measured eHealth literacy using the Transactional eHealth Literacy Instrument (TeHLI) of Paige et al [17]. The TeHLI previously underwent a rigorous instrument development and testing procedure, yielding 4 dimensions consistent with the TMeHL [19]. The TeHLI was selected for this study because of its focus on transactional exchanges within the eHealth domain and because it extends beyond other previously established eHealth measures such as the eHealth Literacy Scale (eHEALS) [17]. Although eHEALS is popular and psychometrically sound [19,31], scholars have noted its limitations in content validity informed by the evolving social possibilities created by eHealth technology, in addition to the lack of correlation between eHEALS scores and enacted task performance pertaining to web-based health information seeking [17]. It also does not sufficiently address the critical and communicative skills included in accurately assessing eHealth literacy [32]. Rather than acting as a static competency, eHealth is fluid and dynamic in nature, which allows individuals opportunities to manage communicative interactions in numerous contexts from multiple sources [33]. This ability to appraise, evaluate, and exchange information between web-based sources is inherently transactional, and this continuous process influences individuals’ relational and cultural contexts and acts as an arbiter of social exchange [17,34]. Norman [32] recommended that eHealth literacy instruments should expand beyond the eHEALS to measure the transactional features afforded by eHealth, and the TeHLI accomplishes this.

The multidimensional TeHLI includes 18 items anchored on a 5-point Likert-type scale, ranging from 1 (strongly disagree) to 5 (strongly agree). The instrument measures four competencies: (1) functional (eg, “I can summarize basic health information from the internet in my own words”); (2) communicative (eg, “I have the skills I need to talk about health topics on the internet with multiple users at the same time”); (3) critical (eg, I can tell when health information on the internet is fake”); and (4) translational (eg, “I can use the internet as a tool to improve my health”). The internal consistency of data from each dimension was sufficient for patients (Cronbach α=.85-.87) and surrogate seekers (Cronbach α=.83-.91). We also sought to understand how patients and surrogate seekers appraise web-based health information. A single, open-ended item was included in the survey asking, “When you found information online, how did you decide if the information was credible?”

### Statistical Analysis

This mixed methods study used a convergent parallel design, in which the quantitative and qualitative data collection occurred concurrently [35]. Per the convergent parallel design, we used the quantitative and qualitative results to compare findings to yield a more holistic understanding of the data [35]. Convergent designs aim to obtain different yet complementary data on the same topic, assist in producing rigorous scholarship through the independent collection of data, and enhance the subsequent comparison and integration of the results of each method. We generated a series of descriptive statistics to summarize the sociodemographic characteristics of the individuals with cancer and caregivers who participated in the survey. We also conducted a 2-tailed independent samples *t* test to examine if the age differed by group, and we conducted chi-square analyses to detect group differences in sex, race, education, and marital status. To test hypothesis 1, we conducted 4 hierarchical multiple linear regression analyses. In step 1, we entered sociodemographics that were empirically shown to correlate with each eHealth literacy competency. In step 2, we estimated whether a difference in each eHealth literacy score existed between individuals with cancer and surrogate seekers while adjusting for sociodemographics in step 1. Variance inflation factors and collinearity were also monitored. Statistical significance was detected at *P*=.05 or lower, and we reported 95% CIs.

As part of the survey, an open-ended question (When you found information online, how did you decide if the information was credible?) was included to investigate how participants determine whether the health information found on the web was credible. Using a content analysis method, we first completed an inductive open-coding process to determine common themes among both respondent populations [36]. Through this procedure, the identification of keywords and commonly discussed content assisted in the development of 8 codes. To understand which action words (or operational behaviors and skills) were used by participants to evaluate web-based cancer information, we extracted the verbs used in their self-reflective procedure. We compared these action words to those reported in a concept analysis of eHealth literacy definitions, models, and measures to inform the most recent definition of eHealth literacy [17]. Table 1 includes the codebook, which went through several training iterations with 2 researchers who coded independently. An acceptable level of intercoder reliability was established (Cohen κ=0.73) after 2 coders independently coded 20% of the data set. A single coder evaluated the remaining data set and determined coder consensus when necessary. A series of chi-square analyses was conducted to examine differences in credibility appraisal (ie, determined source credibility and determined channel credibility) of web-based health information according to patient and surrogate seeker status.

As a final step, integration of the data occurred by merging the quantitative results with the qualitative results [35]. Through triangulation, we compared the results of each data set, quantitative and qualitative, to draw the conclusions set forth in in the results presented [37].

**Table 1:** Codes, operational definitions, and examples to guide open-ended responses.

Code	Definition	Respondent quotes
Determined channel credibility	This code should be used when the respondent answers the question by saying they looked for credible websites and paid attention to where the information was coming from such as Mayo Clinic or a .gov or .edu website. If the respondent double-checked information, read reviews, or trusted the website, these answers could fall under this category as well.	Published in a reputable peer-reviewed journal It came from a credible source like a journal or medical center Study was done by a credible medical institution Research or credible sample size or investigators By whom it was provided I made sure the website is accredited
Determined source credibility	This code should be used when the respondent answers the question by saying they looked up to see who the author was and if they were credible.	I noted the author and the credibility of the institution which it represented I had to x-ref dates and study authors to see what was most current, who was still strong in the field, etc. I looked at the credentials of the author
Checked citations for scientific support	This code should be used when the respondent answers the question by saying they checked the resources of the information to see if it was coming from a credible source. An example of this could be if the respondent looks at the resource the information was taken from, checked the sources at the bottom of the website, age of the article, etc.	I looked at the resources the information came from I checked the sources at the bottom of the website Age of article references Researched the sources listed Looked for citations from doctors at the bottom Source references
Cross-referenced content with other web-based sources	This code should be used when the respondent answers the question by saying they looked at or researched several different sources or web pages to determine if the web-based health information was consistent with each other.	How frequently it was repeated in both articles and university-based publications Cross-referencing multiple sites I checked additional websites to compare information as accurate
Cross-referenced content with recommendations from health care	This code should be used when the respondent answers the question by saying they researched health information on the web to confirm that it aligned with what their clinician recommended. This code can also be used when a patient or caregiver confirms information found on the web between 2 websites.	Compared information with what I had gotten from my medical source Seemed like it was in line with what my health care clinician told me Compared with information I received from my physician and medical team
Discussed content with a health care clinician	This code should be used when the respondent answers the question by saying they discussed the information they found on the web with their health care clinician to check its credibility.	I reviewed my symptoms with the information provided, then discussed the symptoms and information with my physician I asked my doctor about it
Miscellaneous	This code should be used when the respondent answers the question with a response that does not fit into any other predefined reason or cannot be explicitly categorized.	Yes it seemed helpful and made me make a doctor appointment Asked a family member It sounded reasonable with what I knew already I trust hospital information
Uncodeable	This code should be used when the respondent answers the question with a response that does not pertain to the information asked. An example of this could be they did not answer the question correctly or provided information that is not relevant to this data.	Not sure Yes I did Yes Had way to know just had to trust reparation Looks real if all of them about what I think

### Ethical Considerations

This study received institutional review board approval from the University of Florida (IRB#201802322). Each participant completed a waiver of informed consent providing them with clear expectations of what the study entailed and provided their consent before participating. Any identifiable respondent information was anonymized according to ethical privacy standards.

## Results

### Sample Characteristics

A total of 303 participants responded to the survey. A small proportion (n=21, 6.9%) of the participants reported not searching for web-based cancer information in the past 6 months and were excluded from the analyses. The final sample consisted of 282 participants, which included individuals with cancer (n=185, 65.6%) and surrogate seekers (n=97, 34.4%).

Tables 2 and 3 show the sociodemographics of the sample, separated into individuals with cancer and surrogate seeker groups. Individuals with cancer and surrogate seekers were predominantly White (individuals with cancer: 142/185, 76.7% and surrogate seekers: 69/97 71%), female (individuals with cancer: 105/185, 56.8% and surrogate seekers: 65/97, 67%), college educated (individuals with cancer: 100/185, 54.1% and surrogate seekers: 58/97, 59%), married (individuals with cancer: 105/185, 56.8% and surrogate seekers: 47/97, 48%), and from the southeastern region of the nation (individuals with cancer: 143/185, 77.3% and surrogate seekers: 77/97, 79%). The sample was also predominantly non-Hispanic, with only 7 (2.5%) participants identifying as Hispanic. The most common individual with cancer surrogate seekers identified themselves as seeking information for was either a friend (20/97, 21%) or a spouse (13/97, 13%). Multimedia Appendix 2 shows the surrogate seeker types in detail. Age (mean 55.47, SD 15.10; range 20-88 years) was the only sociodemographic variable that varied according to patient and surrogate seeker status. An independent sample 2-tailed *t* test showed that individuals with cancer reported an older age (mean 57.44, SD 14.29 years) than surrogate seekers in this sample (mean 51.72, SD 16.0; *t*_234_=2.80; *P*=.01, 95% CI 1.70-9.75).

**Table 2:** Sociodemographic characteristics (patient sample, N=185).

Variables		Values
Age, mean (SD)		57.44 (14.29)
**Sex, n** **(%)**		
	Male	50 (27)
	Female	105 (56.8)
	Intersex	1 (0.5)
	Missing	29 (15.7)
**Race, n** **(%)**		
	White	142 (76.8)
	Hispanic or Latino	4 (2.2)
	Black or African American	8 (4.3)
	Other	2 (1.1)
	Missing	29 (15.7)
**Education, n (%)**		
	Less than high school	2 (1.1)
	High school or General Education Development	15 (8.1)
	Some college	32 (17.3)
	Completed college	45 (24.3)
	Completed some postgraduate	10 (5.4)
	Master’s degree	31 (16.8)
	Other advanced degree beyond master’s	14 (7.6)
	Missing	36 (19.5)
**Marital status, n (%)**		
	Single	43 (23.2)
	Partnered	9 (4.9)
	Married	105 (56.8)
	Missing	67 (36.2)
**Geographic region, n (%)**		
	Midwest	0 (0)
	Northeast	7 (3.8)
	Southeast	143 (77.3)
	Southwest	6 (3.2)
	West	0 (0)
	Missing	29 (15.7)
**Type of cancer, n (%)^a^**		
	Breast	39 (26.4)
	Skin (squamous cell carcinoma, basal cell carcinoma, melanoma, and Merkel cell)	26 (17.6)
	Blood (leukemia, lymphoma, and myeloma)	12 (6.6)
	Lung	10 (6.8)
	Thyroid	8 (5.4)
	Prostate	6 (4.1)
	Colon and rectal	5 (3.4)
	Non-Hodgkin lymphoma	4 (2.7)
	Ovarian	4 (2.7)
	Pancreatic	4 (2.7)
	Endometrial	2 (1.4)
	Esophageal	2 (1.3)
	Head and neck	2 (1.4)
	Kidney	2 (1.3)
	Mesothelioma	2 (1.4)
	Parotid gland	2 (1.3)
	Throat	2 (1.4)
	Bladder	1 (0.7)
	Bone	1 (0.7)
	Brain	1 (0.7)
	Fallopian tube	1 (0.7)
	Lymphedema	1 (0.7)
	Mesenteric	1 (0.7)
	Sarcoma	1 (0.7)
	Synovial	1 (0.7)
	Testicular	1 (0.7)
	Uterine	1 (0.7)
	Miscellaneous or other	6 (4.1)

^a^Types of cancers (n=148). Individuals with cancer may have reported >1 cancer type. Segments of both patients and surrogate seekers did not provide cancer type.

**Table 3:** Sociodemographic characteristics (surrogate seeker sample, N=97).

Variables			Values
Age (years), mean (SD)			51.72 (19.99)
**Sex, n** **(%)**			
	Male	17 (18)	
	Female	65 (67)	
	Missing	15 (15)	
**Race, n** **(%)**			
	White	69 (71)	
	Hispanic or Latino	3 (3)	
	Black or African American	4 (4)	
	Native American or American Indian	1 (1)	
	Asian or Pacific Islander	1 (1)	
	Other	4 (4)	
	Missing	15 (15)	
**Education, n (%)**			
	Less than high school	1 (1)	
	High school or General Education Development	6 (76)	
	Some college	13 (13)	
	Completed college	41 (42)	
	Completed some postgraduate	2 (2)	
	Master’s degree	12 (12)	
	Other advanced degree beyond master’s	3 (3)	
	Missing	19 (20)	
**Marital status, n (%)**			
	Single	31 (32)	
	Partnered	4 (4)	
	Married	47 (48)	
	Missing	15 (15)	
**Geographic region, n (%)**			
	Midwest	0 (0)	
	Northeast	1 (1)	
	Southeast	77 (79)	
	Southwest	3 (3)	
	West	0 (0)	
	Missing	16 (16)	
**Type of cancer, n (%)^a^**			
	Breast	15 (18)	
	Skin (squamous cell carcinoma, basal cell carcinoma, melanoma, and Merkel cell)	9 (11)	
	Colon and rectal	9 (11)	
	Brain	7 (8)	
	Prostate	6 (7)	
	Blood (leukemia, lymphoma, and myeloma)	4 (3)	
	Lung	4 (3)	
	Ovarian	4 (3)	
	Pancreatic	4 (3)	
	Bladder	3 (4)	
	Liver	2 (2)	
	Kidney	2 (2)	
	Oral	2 (2)	
	Uterine	2 (2)	
	Stomach	2 (2)	
	Head and neck	1 (1)	
	Endometrial	1 (1)	
	Esophageal	1 (1)	
	Appendix	1 (1)	
	Bone	1 (1)	
	Unknown (cannot remember, not sure yet, and unknown)	3 (4)	

^a^Types of cancers (n=83). Surrogate seekers may have reported >1 cancer type. Segments of both patients and surrogate seekers did not provide the cancer type.

### eHealth Literacy

Tables 4 shows the scores for each eHealth literacy competency. On the basis of a 5-point Likert-type scale, participants reported an above-average eHealth literacy score across all 4 competencies. On average, participants “agreed” that they have the skills needed to successfully access and understand web-based health information (*functional eHealth literacy*) and apply what they learned to their health situation (*translational eHealth literacy*). Participants reported neither agreeing nor disagreeing that they have the skills needed to successfully exchange (*communicative eHealth literacy*) and evaluate (*critical eHealth literacy*) web-based information about cancer. Pearson correlation coefficients report a strong, statistically significant association between scores from each eHealth literacy competency (*r*=0.54-0.70; *P*<.001).

**Table 4:** eHealth literacy competency scores.

Competency	Values, n	Values, mean (SD)	Values, median (range)
Functional eHealth literacy	244	4.06 (0.76)	4.00 (1-5)
Communicative eHealth literacy	239	3.29 (0.91)	3.20 (1-5)
Critical eHealth literacy	238	3.49 (0.75)	3.60 (1.60-5)
Translational eHealth literacy	239	3.98 (0.65)	4.00 (2-5)

### eHealth Literacy Differences Between Individuals With Cancer and Surrogate Seekers

Table 5 shows the results of 4 hierarchical linear regression models that examined the association of respondent type (surrogate seeker vs individual with cancer) with each eHealth literacy competency, adjusting for age, gender, race, education, and marital status. The models were statistically significant for each of the four eHealth literacies: (1) functional (*F_6,226_*=4.91, *P*<.001; *R*^2^=0.12, *R*^2adj^=0.09); (2) communicative (*F_6,226_*=4.47, *P*<.001; *R*^2^=0.11, *R*^2adj^=0.08); (3) critical (*F_6,225_*=4.30, *P*<.001; *R*^2^=0.10, *R*^2adj^=0.08); and (4) translational (*F_6,226_*=2.99, *P*=.01; *R*^2^=0.07, *R*^2adj^=0.05).

Functional (*P*=.51), communicative (*P*=.31), and critical eHealth literacy (*P*=.63) scores did not statistically significantly differ between individuals with cancer and surrogate seekers after adjusting for sociodemographic variables. The results of the final regression model demonstrated a trend between respondent status and translational eHealth literacy but was not statistically significant (β=−0.15, SE 0.09; *P*=.09). This result, which should be interpreted with caution, suggests that individuals with cancer may have higher confidence in applying the information they find on the internet to their own health, than their surrogate seeker counterparts.

We also found statistically significant associations between sociodemographic variables and eHealth literacy competencies in step 1 of the hierarchical linear regression models, whether the participant was an individual with cancer or a surrogate seeker. Identifying as a female and reporting a college education resulted in a positive association with functional eHealth literacy (*F*_5,227_=5.82, *P*<.001; *R*^2^=.11, *R*^2adj^=0.09). In the communicative eHealth literacy model, reporting a younger age and a college education was positively associated with confidence in exchanging web-based health information, (*F*_5,227_=5.15, *P*<.001; *R*^2^=.10, *R*^2adj^=0.08). Similarly, reporting a younger age and a college education and identifying as a female resulted in a positive association with critical eHealth literacy (*F*_5,226_=5.13, *P*<.001; *R*^2^=.10, *R*^2adj^=0.08). Translational eHealth literacy, however, was only statistically significantly associated with having a college education (*F*_5,227_=3.00, *P*=.04 *R*^2^=.06, *R*^2adj^=0.04).

**Table 5:** Regression of surrogate seeker versus patient status on eHealth literacy.

Variable		Functional, β (SE; 95% CI)	Communicative, β (SE; 95% CI)	Critical, β (SE; 95% CI)	Translational, β (SE; 95% CI)
**Step 1**					
	Age (years)	−0.01 (0.01; −0.01 to 0.01)	−0.01 (0.01^a^; −0.02 to −0.01)	−0.01 (0.01^a^; −0.02 to −0.01)	.00 (0.00; −0.01 to 0.00)
	Sex^b^	−0.34 (0.11^c^; −0.55 to −0.13)	−0.19 (0.13; −0.45 to 0.07)	−0.25 (0.11^d^; −0.46 to −0.04)	−0.10 (0.10; −0.29 to 0.09)
	Race^e^	.05 (0.15; −0.25 to 0.35)	−0.11 (0.19; −0.48 to 0.25)	−0.11 (0.15; −0.40 to 0.19)	.19 (0.14; −0.07 to 0.46)
	Education^f^	.41 (0.10 ^g^; 0.21 to 0.61)	.34 (0.13^g^; 0.10 to 0.59)	.27 (0.10^g^; 0.07 to 0.47)	.30 (0.10^g^; 0.12 to 0.48)
	Marital status^h^	.17 (.10; −0.03 to 0.36)	−0.04 (0.12; −0.28 to 0.20)	.08 (0.10; −0.12 to 0.28)	−0.06 (0.09; −0.24 to 0.12)
**Step 2**					
	Respondent^i^	.07 (0.10; −0.13 to 0.26)	.13 (0.12; −0.12 to 0.37)	−0.05 (0.10; −0.24 to 0.15)	−0.15 (0.09^f^; −0.33 to 0.03)

^a^*P*<.01.

^b^Sex (1=male; 0=female).

^c^*P*<.001.

^d^*P*<.05.

^e^Race (1=White; 0=people of color).

^f^Education (1=college educated; 0=less than college educated).

^g^*P*=.09.

^h^Marital status (1=married; 0=not married).

^i^Respondent (1=caregiver; 0=patient).

### Individuals With Cancer and Surrogate Seekers’ Evaluations of Credibility

Tables 6 shows that approximately half of the individuals with cancer (70/169, 41%) and surrogate seekers (37/89, 42%) evaluated the credibility of web-based cancer information according to the channel from where it was disseminated. Chi-square analyses showed that the relationship between individuals with cancer and surrogate seekers using the channel of web-based cancer information to appraise its credibility was not statistically significant (*P*=.98). Examples of common information channels reviewed by individuals with cancer include professional and academic institutions such as the Mayo and Cleveland clinics, the National Institutes of Health and National Cancer Institute, medical universities and websites, as well as physicians, peer-reviewed journals, and “credible” websites with .edu or .gov URLs. Examples reviewed by surrogate seekers include university-based publications, health care clinicians, the Mayo Clinic and National Institutes of Health. A similar code, *determined source credibility*, identified that individuals with cancer (8/169, 4.7%) and surrogate seekers (3/89, 3%) looked up to see who the author of web-based health information was and if they themselves were deemed credible. One person said, “Made sure a medical professional wrote the article” (ID 127; White female, 68 years old, completed some college). These respondents also often looked to see if a health care clinician or physician was cited to determine whether the information provided was credible.

**Table 6:** Frequencies of codes reported by individuals with cancer (n=169) and surrogate seekers (n=89).

Code	Individual with cancer, n (%)	Surrogate seeker, n (%)
Determined channel credibility	70 (41.4)	37 (41.6)
Determined source credibility	8 (4.7)	3 (3.4)
Checked citations for scientific support	5 (2.9)	16 (18)^a^
Cross-referenced content with other web-based sources	52 (30.8)^b^	18 (20.2)
Cross-referenced content with recommendations from clinicians	6 (3.6)	3 (3.4)
Discussed content with clinician	11 (6.5)	5 (5.6)
Miscellaneous	13 (7.7)	6 (6.7)
Not coded	4 (2.4)	1 (1.1)

^a^*P*<.001.

^b^*P*<.10.

One code that reflected a considerable difference between individuals with cancer and surrogate seekers was the mode of appraising credibility by *checking citations for scientific support* of the information. A greater proportion of surrogate seekers (16/89, 18%) than individuals with cancer (5/169, 2.9%) checked for citations in their search for scientific information, (χ^2^_1, N=258_=17.6, *P*<.001). Only 3% (5/169) of individuals with cancer checked the citations of web-based health information to determine whether the content provided was credible, whereas nearly 18% (16/89) of surrogate seekers checked citations and references. One surrogate seeker stated as follows:

I checked the sources at the bottom of the website. If no sources (scholarly websites or government/organization website hosts) were provided, then I did not deem it credible.

Surrogate seeker ID 265; White male, 23 years old, college graduate

Other surrogate seekers said, “I decided if it was credible if I had ample sources and clear answers.” (surrogate seeker ID 275; Hispanic female, 21 years old, completed some college); “There were credible references that were less than five years old.” (surrogate seeker ID 344; Black female, 56 years old, college graduate).

The second most used strategy was *cross-referencing content with other* web-based *sources.* Chi-square analyses revealed a trend toward statistical significance between individuals with cancer and surrogate seekers, showing that individuals with cancer may be more likely to cross-check information with other web-based sources than surrogate seekers (*P*=.07). Approximately 30.7% (52/169) of individuals with cancer cross-referenced materials with additional sources, compared with 20% (18/89) of surrogate seekers. An example of a surrogate seeker response includes, “I looked at the date it was written and compared with other websites to see if it was similar—I looked at WebMD and then looked at Mayo Clinic to confirm accuracy” (surrogate seeker ID 233; White female, 51 years old, college educated). Individuals with cancer expressed similar experiences with cross-referencing multiple websites, such as searching to see if information was repeated on several websites and comparing information from similar web-based sources.

Table 6 provides additional procedures used by individuals with cancer and surrogate seekers to evaluate the credibility of web-based health information; however, they were used less frequently and were not statistically significantly different between individuals with cancer and surrogate seekers. This included cross-referencing content with recommendations from health care clinicians (6/169, 3.6% individuals with cancer vs 3/89, 3% surrogate seekers*; P*=.94); discussing content acquired on the web with a health care clinician was also used (11/169, 6.5% individuals with cancer vs 5/89, 6% surrogate seekers*; P*=.78), in addition to determining source credibility to assist in the credibility appraisal of web-based cancer information (8/169, 4.7% individuals with cancer vs 3/89, 3% surrogate seekers; *P*=.61). The representation for each of these credibility appraisal procedures was relatively small and requires further analysis to determine differences, if any, between individuals with cancer and surrogate seekers.

The action words *looked* (individuals with cancer 22/123, 17.9%; surrogate seekers 17/57, 30%), *compared* (individuals with cancer 17/123, 13.8%; surrogate seekers 8/57, 14%), *read or reading* (individuals with cancer 16/123, 13%; surrogate seekers 7/57, 12%), and *searched or researched or tried to find* (individuals with cancer 11/123, 8.9%; surrogate seekers 7/57, 12%) were used most often among this sample (Table 7). When segmented into the 4 eHealth literacies, surrogate seekers used functional eHealth literacy terminology approximately 56% (32/57) of the time, compared with 43.9% (54/123) of individuals with cancer. Individuals with cancer evaluated web-based health information using a critical eHealth literacy perspective 39.8% (49/123) of the time, whereas surrogate seekers used critical eHealth literacy skills approximately 32% (18/57) of the time. There was a wider range of functional eHealth literacy (individuals with cancer: 54/123, 43.9%; surrogate seekers: 32/57, 56%), action word utterances than critical (*IC:* 49/123, 39.8%; surrogate seekers: 23/57, 40%) communicative (individuals with cancer: 12/123, 9.8%; surrogate seekers: 5/57, 9%), and translational (individuals with cancer: 8/123, 6.5%; surrogate seekers: 2/57, 4%).

**Table 7:** Frequencies of action words reported by individuals with cancer (n=123) and surrogate seekers (n=57).

Action words		Patients, n (%)	Surrogate seekers, n (%)
**Functional eHealth literacy**			
	Looked	22 (17.9)	17 (29.8)
	Reviewed	3 (2.4)	0 (0)
	Read or reading	16 (13)	7 (12.3)
	Gathered	1 (0.8)	1 (1.8)
	Texted	1 (0.8)	0 (0)
	Searched or researched or tried to find	11 (8.9)	7 (12.3)
**Communicative eHealth literacy**			
	Spoke or speaking or discussed	5 (4.1)	1 (1.8)
	Asked	7 (5.7)	4 (7)
**Critical eHealth literacy**			
	Checked or double-checked	9 (7.3)	4 (7)
	Cross-referenced	5 (4.1)	2 (3.5)
	Considered or thinking	6 (4.9)	0 (0)
	Compared	17 (13.8)	8 (14)
	Evaluated or screened	2 (1.6)	0 (0)
	Confirmed or made sure or verified	4 (3.3)	1 (1.8)
	Decided or deemed or noted	4 (3.3)	3 (5.3)
	Assumed	2 (1.6)	0 (0)
**Translational eHealth literacy**			
	Used or using	6 (4.9)	2 (3.5)
	Tried	2 (1.6)	0 (0)

## Discussion

### Principal Findings

The purpose of this study was to evaluate the eHealth literacy of individuals with cancer and surrogate seekers and explore the unique processes each group uses to evaluate web-based cancer information. Functional, communicative, critical, and translational eHealth literacy scores did not statistically significantly differ between individuals with cancer and surrogate seekers; however, we found differences in how individuals with cancer and surrogate seekers determine whether web-based cancer information is credible. This brings into question the validity of the web-based content retrieved from each group and how it influences health decisions, behaviors, and outcomes. The results demonstrate the value of understanding both the skill set and the process by which web-based content is accessed and evaluated before its exchange with others.

We did not find any statistically significant differences between individuals with cancer and surrogate seekers’ confidence in their abilities to access, exchange, evaluate, and act on web-based health information for the purposes of maintaining or improving health. Individuals with cancer and surrogate seekers reported a high degree of confidence in their eHealth literacy across all competencies; however, functional and translational eHealth literacy had a slightly higher average score than critical and communicative eHealth literacy scores.

### Comparison With Prior Work

The findings from this study align with previous literature that showed younger, more educated populations having higher eHealth literacy scores and an increased ease of accessing web-based content, often influenced by the level of use [17,20,38]. As this divergence in groups becomes more apparent, future research may examine the various factors that inhibit the communicative and critical evaluation skills of older groups of individuals with cancer and surrogate seekers.

Heiman et al [39] found that the internet was the third most important source of information for individuals with cancer, preceded only by their oncologist and print media. They also discovered that the biggest concern for individuals with cancer was not being able to differentiate between reliable and unreliable websites when searching for information pertaining to their diagnosis and treatment [39]. Our study assessed exactly how individuals with cancer and surrogate seekers evaluated the credibility of web-based health information, which provides further knowledge and understanding to expand upon previous research.

Regardless of their eHealth literacy skill sets, individuals with cancer and surrogate seekers most often determine credibility according to the channel of the web-based cancer information. This suggests that those seeking cancer information do not see a significant need for corroboration of web-based information if the site publishing it is perceived as credible. Several respondents noted that they determined whether web-based health information was credible based on whether they had visited a website in the past and were already familiar with it. Others determined that the information was credible if the website provided the information that the patient or surrogate seeker was searching for. Similar findings have been discussed in the scoping review by Verm et al [40], which found that eHealth literacy was positively correlated with surrogate seekers’ strategies enacted such as seeking a second opinion, awareness of treatment options, shared decision-making, and trust in the health care system. An important factor to consider, moving forward, when analyzing how people appraise and evaluate the credibility of web-based health information includes several cognitive biases such as confirmation bias.

Confirmation bias, or the phenomena of “seeking or interpreting evidence in ways that are partial to existing beliefs, expectations, or a hypothesis in hand” has the potential to greatly influence the subconscious motivations for seeking web-based health information in patients and supporting network roles [41]. Meppelink et al [42] recently found that individuals with high health literacy in web-based health information seeking tend to select belief-consistent information that is rated as credible, useful, and convincing. People who are prone to confirmation bias are considered to be overconfident in their knowledge and skills in evaluating content [43]. Therefore, purposefully seeking information that confirms and validates prior assumptions could negatively impact a patient’s or surrogate seekers’ understanding of a diagnosis, treatment, and health care management and have detrimental impacts on advocacy skills throughout a health care experience. Future research is needed to examine whether this biased information search is fueled by a lack of knowledge acquisition skills or a degree of managing uncertainty surrounding a cancer diagnosis of themselves or their loved ones.

More surrogate seekers than individuals with cancer reviewed the references, citations, and links provided with web-based health information to determine whether the content was credible. Conversely, more individuals with cancer than surrogate seekers cross-referenced content with other web-based sources to determine its credibility. Individuals with cancer may determine the credibility of information through sheer quantity (ie, how often it is repeated across multiple sources), whereas surrogate seekers may determine its credibility based on scientific support and quality of citations. This distinction in information-seeking behaviors and preferred evaluation methods between individuals with cancer and surrogate seekers is important to examine, as individuals with cancer are prone to misinformation and to consensus effects of information [44]. Simply accessing and confirming that a piece of information is available from more than one location does not guarantee its credibility, and dilemmas such as this could inadvertently perpetuate the spread and use of misinformation pertaining to cancer care. In-depth qualitative inquiry is needed to examine the tendencies of individuals with cancer to acquire information (ie, referring to multiple sources and researching patient experiences) compared with surrogate seekers’ preferences for a more scientifically grounded knowledge base (ie, information channels and confirmation of acquired information).

Identifying the diverse and unique ways in which individuals with cancer and surrogate seekers access, appraise, and evaluate the credibility of web-based cancer information could provide a deeper, more tailored design and evaluation of patient education resources. These resources are developed with the intention of being perceived as credible by each recipient, so having distinctive information on how different groups retrieve this web-based content could help us better appeal to the established behaviors that individuals with cancer and surrogate seekers use to enhance their appraisal and evaluation of the credibility of web-based information. Understanding the process by which individuals with cancer and their surrogate seekers access and evaluate web-based information credibility will further inform how to deliver educational content from varying web-based sources, hopefully increasing both patient and surrogate seekers’ autonomy and self-efficacy throughout their cancer care. While identifying the dynamic ways in which individuals with cancer and surrogate seekers access and appraise web-based cancer information yields important insights into future message design and implementation, understanding the interpersonal contexts of this population is imperative for a more refined understanding of why they execute such skills. Researchers should consider nuances related to psychological and relational factors that affect these appraisal and evaluation skills more deeply, including the level of perceived importance that the patient or surrogate seeker has for receiving credible web-based information, how their personal relationships impact their appraisal and evaluation skills, and how stress levels impact the appraisal and evaluation process.

### Limitations

The limitations of this study include addressing the longitudinal effects of eHealth literacy, assessing contextual factors related to individuals with blood cancer, and examining dyadic groups of individuals with cancer and surrogate seekers. First, this was a cross-sectional study, which poses a challenge given that eHealth literacy is a dynamic skill set that evolves over time, making it difficult to determine large effects from 1 period. Future surveillance research is needed to explore how eHealth literacy in individuals with cancer and surrogate seeker groups changes over time. This study explored eHealth literacy in 2 independent groups of individuals with cancer and surrogate seekers. The TeHLI is a relatively new eHealth literacy measure. Similar to the eHEALS, we recognize that more advanced statistical analyses must be conducted to strengthen evidence for its use (eg, measurement invariance and item response theory). Examining measurement invariance in future studies is particularly important as this statistical test is the only way to confirm whether a latent variable can be truly compared across 2 or more groups. Given the exploratory nature of this study, such tests were not conducted.

This study did not take possible contextual factors for individuals with cancer and surrogate seekers into account, such as date of diagnosis, how recently they received their diagnosis compared with when they searched for web-based health information, or the varying levels of stress experienced when participating in this web-based health information seeking and credibility appraisal process. Assessing these factors in future studies could assist in better understanding the unique circumstances experienced within the population and how these interpersonal factors possibly influence eHealth literacy over the course of their cancer journey.

Most respondents in this study were diagnosed with or had cared for someone with breast, skin, or some type of blood cancer. Although not consistent with national estimates of cancer incidence and prevalence [45], the results are consistent with the estimates reported in the catchment area from which these data were collected [46]. Future research should take a more stratified approach to recruitment for cancer type to ensure that region-specific nuances are identified and controlled. In addition, replicating this study among a sufficient sample of individuals with cancer and surrogate seekers dealing with a specific cancer through subgroup analyses will be important for deepening the understanding of any possible differences in eHealth literacy between individuals with cancer and surrogate seekers. The cumulative sample size for this study was relatively small and was restricted to the southeastern region of the nation. The results were limited to a specific region of the United States, and their generalization should be approached with caution. Future research would also benefit from observing individuals with cancer and surrogate seekers as they evaluate web-based cancer information, rather than relying on self-reported procedures that are prone to reporting biases. Regardless of these limitations, the data were derived from validated, theoretically-driven measures and we used a mixed methods approach to achieve the purpose of this study.

### Practical and Theoretical Implications

Using the TMeHL as a theoretical foundation [17], this study aimed to discern how individuals with cancer and surrogate seekers evaluated the credibility of web-based health information. The TMeHL provided an established intrapersonal skill set pertaining to eHealth literacy, which highlighted several discrete skills that were used to interpret the results. From this, we gathered information that showed that eHealth literacy scores remained similar between individuals with cancer and surrogate seekers. However, these groups use their skills in unique and diverse ways. For example, they weigh the features of web-based health information differently when they appraise and evaluate credibility. These differences include source, channel, scientific references, quantity of web-based sources viewed, etc. The features of critical eHealth literacy were centralized around individuals with cancer and surrogate seekers’ descriptions of how they evaluate web-based cancer information. However, skills relevant to all 4 eHealth literacies represented in the TMeHL were represented across these descriptions, suggesting that these skills function together.

Seeing the variance between individuals with cancer and surrogate seekers in what they deem most important when evaluating the credibility of web-based health information offers several avenues for future research, including exploring the potential barriers each group encounters when searching for and appraising web-based information. According to the TMeHL [17], an individual’s eHealth literacy skill set may evolve to proactively overcome challenges that are persistent in their environment and pose threats to their ability to effectively navigate web-based health information. It may be that individuals with cancer or surrogate seekers may each experience different challenges than the other that must be addressed to properly ascertain the appraisal processes for each group. Psychological and relational considerations may also be incorporated to better distinguish between these groups, such as level of support network load or burden, perceived burden of patients with cancer, and varying degrees of self-efficacy and motivation needed to advocate for the importance of credible web-based cancer information related to their health. Further research should also focus on the clear preferences that individuals with cancer and surrogate seekers have that revolve around using sources versus channels to establish whether information is credible.

When viewed practically, strategic messages that are targeted to a group’s preferred source or channel of information will increase the likelihood of its seeing the information as relevant and credible and have greater potential to better initiate patient and surrogate seeker engagement over the course of one’s own or a loved one’s health care management. The continual advances in computer-assisted technologies make tailoring messages to these variables an important next step, as this level of personalization can not only help enhance the individuals with cancer and surrogate seekers’ web-based health information acquisition experience, but also hold considerable potential to provide pertinent information to these populations. Our study included patients and surrogate seekers, but we did not examine the eHealth literacy skills or information-seeking behaviors of individuals with cancer with their own surrogate seekers. Future research with dyads of individuals with cancer and health care clinicians is needed to determine the value of tailored messages within this context.

### Conclusions

Individuals with cancer and surrogate seekers report similar eHealth literacy levels, but there is evidence that these groups apply unique approaches to evaluating the credibility of web-based health information. The results of this study have important theoretical and practical implications for expanding the understanding and applicability of the TMeHL to inform future message design interventions. Future research is needed to examine how dyads of individuals with cancer and surrogate seekers evaluate web-based health information and the acceptability of collaborative patient with cancer support network dyadic eHealth literacy interventions.

## Appendix

Multimedia Appendix 1 Sociodemographic questions from Health Information National Trends Survey and US Census Bureau.

Multimedia Appendix 2 Surrogate seeker types.

## Abbreviations

eHEALS: eHealth Literacy Scale
REDCap: Research Electronic Data Capture
TeHLI: Transactional eHealth Literacy Instrument
TMeHL: Transactional Model of eHealth Literacy

## Data Availability

The data set from which the results of this study were derived can be obtained from the corresponding author upon request.
