# Supplementary material for: Comparing Transactional eHealth Literacy of Individuals With Cancer and Surrogate Information Seekers: Mixed Methods Study
Source: JMIR Form Res. 2022 Sep 28;6(9):e36714. doi: 10.2196/36714 (PMC9557759; doi:10.2196/36714)
Supplement: Multimedia Appendix 1 [file formative_v6i9e36714_app1.docx]

Appendix 1. Socio-demographic questions from HINTS and U.S. Census Bureau.

| What is your highest level of education? |
| --- |
| What is your race? |
| What is your age? |
| What is your sex? |
| What is your education? |
| What is your marital status? |
| What is your geographic region? |
