# Supplementary material for: Comparing Transactional eHealth Literacy of Individuals With Cancer and Surrogate Information Seekers: Mixed Methods Study
Source: JMIR Form Res. 2022 Sep 28;6(9):e36714. doi: 10.2196/36714 (PMC9557759; doi:10.2196/36714)
Supplement: Multimedia Appendix 2 [file formative_v6i9e36714_app2.docx]

Appendix 2. Surrogate Seeker Types.

| Surrogate Seeker | N (%) |
| --- | --- |
| Friend | 20 (20.26) |
| Spouse | 13 (13.40) |
| Mother/Mother-in-law | 6 (6.19) |
| Daughter | 6 (6.19) |
| Son | 5 (5.15) |
| Acquaintance | 4 (4.12) |
| Brother/Brother-in-law | 4 (4.12) |
| Father/Father-in-law | 3 (3.09) |
| Aunt | 3 (3.09) |
| Miscellaneous | 3 (3.09) |
| Nephew | 2 (2.06) |
| Niece | 2 (2.06) |
| Cousin | 2 (2.06) |
| Grandmother | 2 (2.06) |
| Uncle | 1 (1.03) |
| Granddaughter | 1 (1.03) |
| Sister/Sister-in-law | 1 (1.03) |
| Missing | 19 (19.59) |
| Total | 97 |
